# *Bacillus velezensis* GB03 Modulates Local and Systemic Responses of Sweet Basil to Root Herbivory by *Diabrotica speciosa*

**DOI:** 10.3390/plants15213365

**Published:** 2026-10-30

**Authors:** Jimena Sofía Palermo, Lorena del Rosario Cappellari, Marilina Fernandez, Erika Banchio

**Affiliations:** INBIAS Instituto de Biotecnología Ambiental y Salud, Campus Universitario, CONICET-Universidad Nacional de Río Cuarto, Río Cuarto 5800, Argentinamfernandez@exa.unrc.edu.ar (M.F.)

**Keywords:** plant growth-promoting rhizobacteria, belowground herbivory, plant–microbe–insect interactions, total phenolic content, volatile organic compounds, essential oils

## Abstract

Plants are naturally exposed to multiple biotic interactions that can simultaneously influence their growth, metabolism, and defense responses. Plant growth-promoting rhizobacteria (PGPR) can influence plant growth and defense, yet their role in shaping plant responses to belowground herbivory remains poorly understood. This study examines how inoculation with *Bacillus velezensis* GB03 and root herbivory by *Diabrotica speciosa* affect growth, specialized metabolism, and hormonal signaling in sweet basil (*Ocimum basilicum*), considering responses in both roots and shoots. GB03 promoted plant growth, whereas root herbivory reduced biomass and strongly activated jasmonate-related signaling. Both GB03 inoculation and herbivory altered specialized metabolism, increasing essential oil accumulation, phenolic content, and volatile organic compound emission, although these responses differed between roots and shoots. The combined treatment produced the strongest response for several specialized-metabolism traits, particularly essential oil and eugenol accumulation and shoot phenolic content. Importantly, the effects of herbivory on shoot phenolic accumulation, root volatile emission, and jasmonic acid levels depended on the inoculation status of the plants, indicating that GB03 selectively modified specific responses to root herbivory. These findings reveal a complex interplay between beneficial rhizobacteria and belowground herbivores, in which GB03 not only promotes plant growth and modifies basal metabolism but also reshapes specific local and systemic responses to herbivore attack. Understanding these interactions may contribute to the development of sustainable strategies that exploit beneficial microorganisms to improve plant performance and modulate specialized metabolism in aromatic crops.

## 1. Introduction

Plants are continuously exposed to multiple biotic challenges occurring both above- and belowground and have evolved complex mechanisms to perceive damage and coordinate defensive responses throughout the plant. While plant responses to foliar herbivores have been extensively studied, considerably less is known about the mechanisms triggered by root-feeding insects [1,2,3]. Root herbivory represents a particularly complex challenge because damage occurs in organs directly involved in water and nutrient acquisition and can therefore affect both plant growth and resource allocation [1,4]. Moreover, responses to root herbivores are not restricted to the damaged organ but can extend systemically to aerial organs, altering primary and specialized metabolism and potentially modifying subsequent interactions with other organisms [1,5,6,7]. Such belowground–aboveground signaling allows plants to coordinate local and systemic responses and can lead to substantial changes in defensive chemistry throughout the plant [4,5,7].

Phytohormonal signaling plays a central role in coordinating these responses. Jasmonates are particularly important in plant responses to herbivorous insects, participating in the regulation of herbivore-induced defenses [8,9,10]. However, herbivore responses are not governed by a single hormonal pathway. Salicylic acid (SA) can interact with jasmonate signaling, while abscisic acid (ABA), traditionally associated with abiotic stress responses, can also participate in the regulation of plant defenses against herbivores [11,12,13,14]. The balance and crosstalk among these signaling pathways can influence the magnitude and nature of the metabolic response to herbivore attack [11,12,14,15]. In the case of belowground herbivory, changes initiated in damaged roots may also modify hormonal and metabolic responses in undamaged aerial organs, providing a mechanistic link between root damage and systemic changes in plant defense [1,5,16].

Aromatic plants of the Lamiaceae provide a particularly suitable system for examining these responses because their specialized metabolism is characterized by a high diversity of terpenoids, phenylpropanoids, and other phenolic compounds with ecological functions in plant defense [17,18,19,20,21]. In sweet basil (*Ocimum basilicum*), essential oils (EOs) constitute an important component of specialized metabolism and contain biologically active compounds such as 1,8-cineole, linalool, α-terpineol, and eugenol [22,23,24,25]. Several of these compounds can also be released as volatile organic compounds (VOCs), contributing to the volatile blend emitted by the plant in response to biotic stimuli [26,27,28]. Changes in EO accumulation and VOC emission may therefore represent complementary components of the phytochemical response, reflecting changes in both stored and emitted specialized metabolites. Phenolic compounds constitute another major component of plant specialized metabolism and can contribute to defense through multiple functions associated with plant responses to biotic stress [29,30,31,32].

Plant growth-promoting rhizobacteria (PGPR) add another level of complexity to plant–herbivore interactions. These beneficial microorganisms colonize the rhizosphere and can promote plant growth through mechanisms involving nutrient acquisition, phytohormone modulation, and changes in plant physiology [33,34,35]. Beyond their growth-promoting effects, some PGPR can enhance plant resistance to subsequent biotic challenges through induced systemic resistance (ISR) or by establishing a primed physiological state [36,37,38,39]. Primed plants may respond to subsequent attack with faster, stronger, or qualitatively different defensive responses, often involving changes in hormonal signaling and specialized metabolism [36,40,41]. Importantly, rhizobacterial effects on plant defense may include both constitutive changes occurring before herbivore attack and modifications in the response subsequently elicited by herbivory [38,42,43,44,45].

*Bacillus velezensis* GB03 was originally described as *Bacillus subtilis* GB03 and was subsequently reclassified as *Bacillus amyloliquefaciens* GB03 before its current classification as *B. velezensis* GB03 [46,47]. Previous studies have shown that GB03 can induce systemic physiological and metabolic changes in plants, including modifications in specialized metabolism [48,49,50,51,52]. In aromatic species, inoculation with beneficial rhizobacteria has been associated with increased EO production, changes in EO composition and VOC emission, and modulation of phenolic metabolism [53,54,55]. These effects are particularly relevant because changes in specialized metabolites may influence not only plant physiology but also plant interactions with herbivorous insects [38,43,51,56,57,58]. However, comparatively little is known about how rhizobacterial inoculation modifies the metabolic and molecular responses triggered by belowground herbivory, or whether these effects are expressed similarly in damaged roots and undamaged aerial organs [59,60,61].

Root-feeding insects provide an especially interesting context in which to examine this interaction because herbivore damage and PGPR–plant interactions occur within the same belowground compartment. *Diabrotica speciosa* (Coleoptera: Chrysomelidae) is a polyphagous pest widely distributed in South America and associated with several economically important crops [62,63]. While adults feed on aerial plant organs, larvae develop in the soil and feed primarily on roots [63]. Larval feeding can directly damage the root system and thereby alter plant growth and physiology, while potentially triggering responses that extend to aerial organs [1,7].

In this study, we investigated the effects of root herbivory by *D. speciosa* larvae, inoculation with the PGPR *B. velezensis* GB03, and their combination on the growth and specialized metabolism of *O. basilicum*. While PGPR inoculation and insect herbivory are individually known to influence plant metabolism and defense responses, their combined effects, particularly in the context of belowground herbivory, remain poorly understood. We therefore examined how GB03 inoculation influences the biochemical, molecular, and hormonal responses of basil to root herbivory, considering both root and shoot organs. This integrative approach provides insight into how rhizobacteria modulate local and systemic plant responses to belowground herbivores and contribute to the regulation of specialized metabolism.

## 2. Results

### 2.1. Plant Biomass in Response to PGPR Inoculation and Root Herbivory

Both GB03 inoculation and root herbivory by larvae of *D. speciosa* affected biomass accumulation in *O. basilicum* (Figure 1). GB03 promoted plant growth under non-herbivory conditions, increasing both shoot and root dry weight compared with non-inoculated plants (*p* < 0.05). In contrast, root herbivory reduced biomass in both inoculation conditions (*p* < 0.05).

In non-inoculated plants, herbivory reduced shoot dry weight by approximately 33% relative to undamaged controls (*p* < 0.05), whereas in GB03-inoculated plants the reduction was approximately 27% (Figure 1A). Similarly, root dry weight decreased by approximately 42% in non-inoculated plants and by 38% in inoculated plants following herbivory (*p* < 0.05, Figure 1B). Despite these reductions, shoot and root biomass of inoculated plants subjected to herbivory were comparable to those of non-inoculated plants without herbivory, indicating that the growth-promoting effect of GB03 partially compensated for the biomass loss associated with root herbivory.

### 2.2. GB03 Inoculation and Root Herbivory Increase EO Accumulation

EO accumulation was markedly increased by both GB03 inoculation and root herbivory in shoot and root organs (Figure 2). In shoots, GB03 inoculation and root herbivory increased EO content by approximately 115% and 120%, respectively, compared with untreated control plants (*p* < 0.05) (Figure 2A). Both treatments resulted in similar EO levels, whereas plants exposed to GB03 inoculation combined with herbivory showed the highest accumulation, with an approximately three-fold increase relative to untreated controls and significantly higher EO content than either treatment alone (*p* < 0.05). A similar response was observed in roots. GB03 inoculation and root herbivory increased EO accumulation by approximately 129% and 143%, respectively, compared with untreated controls (*p* < 0.05; Figure 2B). The combined GB03 + herbivory treatment produced the highest EO accumulation, reaching approximately 3.6-fold that of untreated plants (Figure 2B).

### 2.3. EO Composition Is Differentially Modulated by GB03 and Root Herbivory

The major constituents of shoot EO were 1,8-cineole, linalool, α-terpineol, and eugenol, whose accumulation was differentially affected by GB03 inoculation and root herbivory (Table 1). In shoots, 1,8-cineole accumulation increased in response to both GB03 inoculation and root herbivory. GB03 inoculation alone increased its concentration by approximately 50% compared with untreated controls, whereas herbivory resulted in a two-fold increase. The combined treatment produced the highest accumulation, although it did not differ significantly from herbivory alone (*p* < 0.05). Linalool showed a marked increase in response to either GB03 inoculation or root herbivory (*p* < 0.05), with concentrations approximately three-fold higher than in untreated controls. However, combining GB03 inoculation with herbivory did not further increase linalool accumulation, with all three treatments showing comparable levels. α-terpineol also increased in response to both treatments. GB03 inoculation approximately doubled its concentration, whereas herbivory alone and the combined treatment produced the highest levels, approximately three-fold those of untreated controls. Among the major shoot EO constituents, eugenol showed the strongest response. GB03 inoculation more than doubled its accumulation, while root herbivory resulted in an approximately three-fold increase compared with untreated controls (*p* < 0.05). The combination of GB03 inoculation and herbivory produced the highest eugenol accumulation, nearly four-fold that of untreated plants and significantly higher than either treatment alone.

In roots, the major EO constituents were endo-borneol and eugenol (Table 2). Eugenol accumulation was strongly enhanced by both GB03 inoculation and root herbivory, increasing approximately seven- and five-fold, respectively, compared with untreated controls (*p* < 0.05). The combined treatment produced the highest accumulation, reaching approximately 12-fold that of untreated plants and significantly exceeding either treatment alone (*p* < 0.05). In contrast, endo-borneol showed only minor variation among treatments, with the combined GB03 + herbivory treatment differing from herbivory alone, while the remaining treatments showed intermediate and statistically comparable values (*p* > 0.05).

### 2.4. GB03 and Root Herbivory Alter VOC Emission from Shoots and Roots

The effects of GB03 inoculation and root herbivory extended beyond EO accumulation to VOC emission. In shoots, GB03 inoculation markedly increased total VOC emission, reaching approximately 2.6-fold that of untreated control plants (*p* < 0.05) (Figure 3A). In contrast, root herbivory alone did not significantly modify total shoot VOC emission compared with untreated controls (*p* > 0.05). The combined GB03 + herbivory treatment produced the greatest response, with total VOC emission reaching approximately five-fold that of untreated controls and approximately two-fold that observed with GB03 inoculation alone (*p* < 0.05).

A different response pattern was observed in roots. Root herbivory increased total VOC emission by approximately 2.2-fold in non-inoculated plants, while GB03 inoculation also enhanced basal root VOC emission compared with untreated controls (*p* < 0.05) (Figure 3B). However, herbivory did not further increase VOC emission in GB03-inoculated plants, resulting in a significant interaction between inoculation and root herbivory. Thus, the effect of root herbivory on total root VOC emission depended on the inoculation status of the plants.

Among the major VOCs emitted from shoots, 1,8-cineole, linalool, α-terpineol, and eugenol showed distinct responses to GB03 inoculation and root herbivory (Table 3). Linalool emission increased approximately 2.5-fold following GB03 inoculation and reached its highest level under the combined treatment, with an approximately 4.3-fold increase relative to untreated controls (*p* < 0.05). A particularly marked response was also observed for α-terpineol, whose emission increased approximately 3.5-fold under herbivory and eight-fold when GB03 inoculation and herbivory were combined (*p* < 0.05). Eugenol emission increased approximately five-fold following GB03 inoculation, but no further increase occurred when herbivory was combined with inoculation (*p* > 0.05). For 1,8-cineole, the combined treatment resulted in an approximately two-fold increase relative to untreated controls and was the only treatment that differed significantly from the control (*p* < 0.05).

In roots, endo-borneol and eugenol were the major VOCs detected (Table 4). Root herbivory increased endo-borneol emission approximately three-fold in non-inoculated plants (*p* < 0.05), while GB03 inoculation produced a comparable increase. Combining GB03 inoculation with herbivory did not further enhance endo-borneol emission (*p* > 0.05). Eugenol emission increased approximately 1.8-fold following GB03 inoculation compared with untreated controls (*p* < 0.05), whereas root herbivory alone showed an intermediate response. The combined treatment showed eugenol emission comparable to that observed with GB03 alone (*p* > 0.05).

### 2.5. Total Phenolic Content Shows Organ-Specific Responses to Inoculation and Herbivory

Total phenolic content (TPC) showed distinct responses to GB03 inoculation and root herbivory in shoot and root *organs* (Figure 4). In shoots, GB03 inoculation increased TPC by approximately 33% compared with untreated control plants (*p* < 0.05), whereas root herbivory alone did not significantly affect phenolic accumulation (*p* > 0.05). In GB03-inoculated plants, however, subsequent root herbivory further increased shoot TPC by approximately 33% relative to GB03 alone (*p* < 0.05), resulting in the highest phenolic content among treatments, approximately 76% above untreated controls (Figure 4A). This differential response resulted in a significant interaction between GB03 inoculation and root herbivory.

In roots, both GB03 inoculation and root herbivory increased phenolic accumulation. GB03 inoculation nearly doubled root TPC, while herbivory alone increased it by approximately 68% relative to untreated controls (*p* < 0.05). The combined GB03 + herbivory treatment produced the highest root TPC, reaching approximately 2.7-fold that of untreated plants (*p* < 0.05) (Figure 4B).

### 2.6. GB03 Inoculation and Root Herbivory Differentially Regulate EGS and C4H Expression

To determine whether the observed changes in specialized metabolism were accompanied by transcriptional regulation, the relative expression of eugenol synthase *(EGS)* and cinnamate 4-hydroxylase *(C4H*), genes associated with eugenol and phenylpropanoid biosynthesis, was analyzed in leaves (Figure 5). The expression of *EGS* varied depending on the treatment applied. GB03 inoculation significantly upregulated *EGS* expression compared with untreated control plants (*p* < 0.05) (Figure 5). Root herbivory by *D. speciosa* also significantly increased *EGS* expression in non-inoculated plants (*p* < 0.05). When GB03 inoculation and root herbivory were combined, *EGS* expression increased further, reaching the highest level observed among all treatments and being significantly higher than that detected in plants exposed to either GB03 inoculation or root herbivory alone (*p* < 0.05). A different response pattern was observed for *C4H*. GB03 inoculation significantly increased *C4H* expression compared with untreated control plants (*p* < 0.05) (Figure 5). Root herbivory alone produced a smaller increase, whereas plants exposed to the combined GB03 + herbivory treatment showed expression levels comparable to those observed in plants inoculated with GB03 alone. Thus, *C4H* expression was significantly higher in GB03 and GB03 + herbivory plants than in untreated controls, but herbivory did not further increase *C4H* expression in GB03-inoculated plants (*p* > 0.05).

### 2.7. Root Herbivory Strongly Modifies the Endogenous Phytohormone Profile

To further understand the regulation of plant responses associated with the observed changes in specialized metabolism, we quantified the endogenous levels of phytohormones in leaves. The jasmonates evaluated—jasmonic acid (JA), jasmonoyl-isoleucine (JA-Ile) and 12-oxo-phytodienoic acid (OPDA)—consistently increased following *D. speciosa* root herbivory (Figure 6A–C). In non-inoculated plants, herbivory increased JA approximately eight-fold, JA-Ile four-fold, and OPDA 1.4-fold relative to undamaged controls (*p* < 0.05). Herbivory also increased these compounds in GB03-inoculated plants, with JA, JA-Ile, and OPDA increasing approximately 2.5-, 3-, and 1.7-fold, respectively, compared with inoculated plants without herbivory (*p* < 0.05). However, while no significant inoculation × herbivory interaction was detected for JA-Ile or OPDA (*p* > 0.05), a significant interaction was observed for JA (*p* < 0.05), indicating that GB03 modified the magnitude of JA induction following root herbivory. Accordingly, the highest JA levels were observed in non-inoculated plants subjected to herbivory. SA showed a different response pattern (Figure 6D). GB03 inoculation increased basal SA levels compared with untreated controls (*p* < 0.05), while root herbivory increased SA approximately two-fold in non-inoculated plants (*p* < 0.05). However, herbivory did not further increase SA in GB03-inoculated plants (*p* > 0.05), and no significant inoculation × herbivory interaction was detected (*p* > 0.05). In contrast, ABA decreased markedly following root herbivory (Figure 6E), with reductions of approximately 59% and 62% in non-inoculated and GB03-inoculated plants, respectively, relative to their corresponding non-herbivory controls (*p* < 0.05). ABA levels were not significantly affected by GB03 inoculation (*p* > 0.05).

### 2.8. Integrated Multivariate Response to GB03 Inoculation and Root Herbivory

Principal component analysis (PCA) integrating growth, hormonal, molecular, and specialized-metabolism variables separated the four experimental conditions (Figure 7). The first two principal components explained 92.8% of the total variance, with PC1 accounting for 62.6% and PC2 for 30.2%. PC1 mainly separated untreated control plants from plants exposed to GB03 inoculation and/or root herbivory. Positive PC1 scores were associated with variables related to specialized metabolism, including shoot and root total phenolic content (STPC and RTPC), shoot and root VOC emission (SVOC and RVOC), shoot and root essential oils (SEO and REO), and expression of EGS and C4H. Plants subjected to the combined GB03 + root herbivory treatment were positioned in the positive region of PC1 and were closely associated with most of these metabolic and molecular variables. PC2 further distinguished the responses to the individual treatments. GB03-inoculated plants without herbivory were positioned in the upper region of the PCA and were associated with shoot and root dry weight (SDW and RDW) and ABA. In contrast, non-inoculated plants subjected to root herbivory were positioned in the lower-right quadrant, in the direction of the jasmonate-related hormones. The combined GB03 + root herbivory treatment was separated from both individual treatments and showed the closest association with phenolic accumulation, EO and VOC production, and EGS and C4H expression.

## 3. Discussion

The present study shows that *B. velezensis* GB03 modifies the physiological and metabolic status of *O. basilicum* and influences the responses elicited by belowground herbivory by *D. speciosa*. GB03 promoted shoot and root growth and enhanced several components of specialized metabolism in the absence of herbivory, whereas root feeding reduced plant biomass and induced marked metabolic and hormonal responses in both roots and shoots. When GB03 inoculation and herbivory occurred together, several specialized-metabolism traits reached their highest levels, revealing that the effects of these two biotic factors extend across different organs and levels of plant response.

GB03 promoted both shoot and root growth, consistent with the well-established growth-promoting capacity of this strain and with previous studies in aromatic plants, including *O. basilicum* [48,49,50,53]. In contrast, root feeding by *D. speciosa* reduced shoot and root biomass, as expected from damage to organs directly involved in water and nutrient acquisition [1,2,64]. Although herbivory reduced biomass in both inoculated and non-inoculated plants, damaged GB03-inoculated plants maintained biomass values comparable to untreated non-inoculated controls. Thus, GB03 did not prevent the growth cost imposed by herbivory, but the greater biomass of inoculated plants partially compensated for herbivore-associated losses [38,44,65,66].

Beyond its effects on growth, GB03 strongly affected basil specialized metabolism. Inoculation increased EO accumulation in both shoots and roots, in agreement with previous reports showing that PGPR can stimulate EO production in aromatic plants, including *Mentha piperita* [55,67], *Origanum* spp. [49,53], *Tagetes minuta* [68], and *O. basilicum* [51]. Similar increases have also been reported in basil and *Satureja hortensis* inoculated with combinations of arbuscular mycorrhizal fungi and PGPR [69], although this response is not universal among aromatic plant–microorganism associations [70]. Root herbivory by *D. speciosa* also enhanced EO accumulation in both organs. Increased EO production following chewing herbivory has previously been reported in *M. piperita* attacked by *Rachiplusia nu* [55] and in *O. basilicum* damaged by the foliar herbivore *Spodoptera frugiperda* [51]. The present findings extend these observations to a root-feeding herbivore, an interaction for which considerably less information is available. Plants exposed to both GB03 and *D. speciosa* showed the highest EO accumulation in shoots and roots. However, because the effects of inoculation and herbivory on total EO were statistically independent, the particularly high values observed under the combined treatment should not be interpreted as a synergistic response.

The response of individual EO constituents provides additional insight into these metabolic changes. In shoots, the major compounds were differentially affected by GB03 inoculation and root herbivory. Similar changes in these major constituents have previously been observed in basil following GB03 inoculation and foliar herbivory [51], while herbivore-induced changes in terpenoid composition have also been reported in *Origanum vulgare* and *Minthostachys mollis* [71,72,73]. Among the compounds analyzed here, eugenol showed a particularly strong response, reaching its highest concentration when inoculation and herbivory occurred together. This response was especially pronounced belowground, where the combined treatment produced an approximately 12-fold increase in root eugenol relative to untreated plants.

The occurrence of eugenol in basil roots is particularly relevant for interpreting this response. Although basil EO is mainly synthesized and stored in glandular trichomes in aerial organs [74], roots possess their own biochemical machinery for eugenol biosynthesis. Reddy et al. [75] identified eight eugenol synthases (ObEGS1–8) in basil and showed that, whereas ObEGS1 is predominantly expressed in glandular trichomes, ObEGS4 shows its highest expression in roots. The same study demonstrated that eugenol production in roots can be induced by methyl jasmonate and other biotic stress-related signals, supporting the existence of a stress-responsive biosynthetic pathway in this organ [75]. More recently, Hirose et al. [76] provided evidence that eugenol detected in roots may have a dual origin, resulting from both long-distance transport from aerial organs through the phloem and local biosynthesis in roots. Thus, the pronounced accumulation of eugenol observed belowground following GB03 inoculation and *D. speciosa* herbivory could involve changes in both local biosynthesis and whole-plant allocation of this phenylpropanoid. The strong activation of jasmonate-related signaling following root herbivory observed in the present study provides an additional mechanistic context for this response. Because EGS expression was analyzed only in leaves, future studies should evaluate root-specific isoforms to determine whether local biosynthesis contributes to the strong accumulation of eugenol in roots.

Changes in EO accumulation were accompanied by modifications in VOC emission, although both responses were not necessarily parallel. Several major EO constituents were also detected in the emitted volatile blend. GB03 increased basal VOC emission from both organs, consistent with previous reports in *M. piperita* inoculated with GB03 and in basil exposed to bacterial volatiles produced by this strain [48,54].

The response to *D. speciosa* feeding was strongly organ-dependent. Root herbivory increased VOC emission belowground but, by itself, did not significantly alter total emission from shoots. Herbivore-induced changes in root volatiles have been described in other belowground systems. For example, maize roots attacked by *Diabrotica virgifera* modify their volatile blend, including the release of (E)-β-caryophyllene and other compounds involved in belowground signaling and indirect defense (Rasmann et al. 2005; Robert et al. 2012) [77,78]. In roots, herbivory increased VOC emission in non-inoculated plants, whereas no further increase occurred in plants already inoculated with GB03, suggesting that bacterial colonization had established a distinct volatile-metabolic state in this organ. A different pattern was observed systemically. Although *D. speciosa* feeding alone did not significantly increase total shoot VOC emission, plants simultaneously exposed to GB03 and root herbivory showed the highest aerial emission. Changes in individual compounds, particularly linalool and α-terpineol, further demonstrate that a belowground herbivore can modify volatile metabolism in undamaged aerial organs. Such systemic changes are consistent with evidence that root herbivory can alter aboveground chemical signaling and thereby influence interactions occurring in other parts of the plant [5,6,78].

Phenolic metabolism was also strongly affected by the treatments. GB03 increased TPC in both shoots and roots, consistent with previous reports of enhanced phenolic accumulation following PGPR inoculation in aromatic plants [54,68,79]. Root herbivory produced an organ-dependent response: phenolic accumulation increased in roots regardless of inoculation, whereas in shoots the strongest response occurred in GB03-inoculated plants exposed to *D. speciosa*. Thus, GB03 not only increased basal phenolic accumulation but also modified the systemic phenolic response of basil to root herbivory. Similar herbivore-induced changes in phenolic metabolism have been reported in *M. piperita* and other plant–herbivore systems [44,55].

Changes in phenolic and EO metabolism were accompanied by transcriptional responses in the phenylpropanoid pathway. *EGS* expression increased in response to both GB03 and root herbivory and was highest under their combined treatment, broadly paralleling the accumulation of eugenol. In contrast, *C4H* responded mainly to GB03, indicating that transcriptional responses did not fully mirror the changes in total phenolic accumulation.

Hormonal changes provide a possible regulatory context for these metabolic responses. Root herbivory strongly activated jasmonate signaling, increasing JA, JA-Ile, and OPDA, consistent with the central role of this pathway in responses to tissue-damaging herbivores [8,12,80]. Interestingly, JA induction was lower in GB03-inoculated plants than in non-inoculated plants following herbivory, suggesting that GB03 did not simply amplify the jasmonate response but rather modified the hormonal context in which basil responded to root damage. Together with the changes observed in SA and ABA, this hormonal reconfiguration may contribute to the altered accumulation and emission of specialized metabolites observed in inoculated and herbivore-damaged plants. Rather than reflecting a direct correspondence between individual hormones and metabolites, these results suggest that GB03 modifies the regulatory network underlying basil responses to belowground herbivory.

Previous studies using the same *O. basilicum*–GB03 system indicate that the metabolic and hormonal responses to herbivory vary according to the feeding mode and site of attack, including foliar chewing, phloem feeding, and root herbivory [51,52,56,81]. Importantly, GB03-induced changes in basil phytochemistry may have biological consequences for herbivores. In previous studies, *S. frugiperda* larvae feeding on GB03-inoculated basil showed reduced growth and food-conversion efficiency, prolonged development, reduced adult emergence, and preference for non-inoculated leaves [56]. In this context, the changes in EO accumulation and composition, particularly the increased accumulation of eugenol, together with changes in phenolic compounds and VOC emission observed here, may have implications for plant defense. Several of these metabolites have documented insecticidal, antifeedant, or deterrent activities [82,83,84,85]. However, direct assays of *D. speciosa* preference, feeding, and performance will be necessary to determine whether these phytochemical changes translate into increased resistance to this root herbivore.

PGPR-induced systemic resistance is frequently associated with a primed state in which previous microbial exposure modifies the capacity of plants to respond to a subsequent challenge [36,40,41,86]. Some responses observed here, particularly the enhanced shoot phenolic accumulation following root herbivory in GB03-inoculated plants, are compatible with a priming-like response. However, GB03 did not uniformly enhance all herbivore-induced responses. The diversity of metabolic and hormonal patterns observed therefore suggests selective modulation of plant responses by GB03 rather than a generalized priming of basil defense.

GB03 appears to establish a distinct physiological and metabolic state that modifies how basil responds to subsequent herbivory. In the present study, this modulation extended across roots and shoots, linking belowground herbivory with systemic changes in specialized metabolism and hormonal signaling. Such context-dependent responses may be particularly relevant in aromatic crops, where beneficial rhizobacteria can simultaneously influence plant performance and specialized metabolites of both ecological and economic importance.

## 4. Materials and Methods

### 4.1. Bacterial Strains, Culture Conditions and Media

The PGPR strain utilized in this study was *B. velezensis* GB03. It was cultured on Luria–Bertani (LB) medium (Laboratorios Britania S.A., Buenos Aires, Argentina) and preserved in nutrient broth with 15% glycerol at −80 °C for long-term storage. For experimental purposes, the bacteria were cultured on LB nutrient agar. Single colonies were then transferred to 100 mL flasks containing the LB medium and grown aerobically on a rotating shaker (150 rpm) for 24 h at 28 °C. The resulting bacterial suspension was diluted in sterile saline solution (0.9% sodium chloride, NaCl) to achieve a final concentration of 10^8^ colony-forming units (CFU) per milliliter.

### 4.2. Seed Sterilization and Plant Cultivation

Seeds of *O. basilicum* var. Genovese (Florensa Argentina S.A., Córdoba, Argentina) were surface sterilized by soaking for 2 min in 70% (*v*/*v*) ethanol and for 20 min in 1% (*v*/*v*) sodium hypochlorite. After this, they were thoroughly rinsed 4 times with sterile distilled water and placed in plastic pots filled with sterilized vermiculite. Following a 15-day period, the plantlets were transplanted into larger plastic pots (12 cm × 22 cm) filled with sterilized vermiculite. They were grown in a growth chamber under controlled conditions of light (16/8 h light/dark cycle), temperature (22 ± 2 °C), and relative humidity (~70%) and watered every week with 20 mL of Hoagland solution per pot.

### 4.3. Insect Rearing: Diabrotica Speciosa

Founder adults were collected from the flowers of squash plants (*Cucurbita maxima*) grown in the peri-urban horticultural belt of Río Cuarto, Córdoba province, Argentina, during January and February. Insects were subsequently reared in the laboratory following Cabrera Walsh [87]. Adults were kept in entomological cages in a growth chamber at 24–28 °C under a 16:8 h photoperiod, provided with water-soaked cotton pads and fed daily with lettuce (*Lactuca sativa*) leaves wrapped in moistened black cotton cloth, which also served as an oviposition substrate. Oviposition cloths were removed from the cages daily, and eggs were dislodged with a water jet into a mesh-covered beaker. Maize seedlings (non-transgenic “pisingallo” popcorn) were grown in pots containing autoclaved sandy loam soil and peat (1:1 *v*/*v*, 40% moisture); seeds were surface-sterilized in 1% sodium hypochlorite for 2 h and soaked in water for 8–12 h before sowing. The collected eggs were then placed on the soil surface of pots containing recently emerged maize seedlings and incubated at 22 ± 2 °C, ~70% relative humidity and a 16:8 h (light:dark) photoperiod until hatching, which occurred after approximately 15 days. Neonate larvae moved into the soil and fed directly on the seedling roots, so no handling of the larvae was required. Ten days after hatching, seedlings were transferred to larger containers to allow continued plant growth and the completion of larval development. Emerging adults were collected and transferred to stock cages to maintain the colony. Third-instar larvae obtained through this procedure were used in the experiments.

### 4.4. Experimental Design and Herbivory Treatment

At 45 days post-inoculation (dpi), *O. basilicum* plants were subjected to herbivory by placing five third-instar *D. speciosa* larvae on the substrate surface around the stem of each plant. After seven days of herbivory, larvae were removed and plants were immediately harvested. For biomass determination, shoots and roots of 20 plants per treatment were separated, dried at 60–70 °C until constant weight, and weighed. In the remaining plants, leaves and roots were collected separately, frozen in liquid nitrogen and stored at −80 °C until analysis. EO content and TPC were determined in both leaves and roots, VOCs released from roots were analyzed in root samples, and phytohormones and gene expression were analyzed in leaves only (see Section 4.5, Section 4.6, Section 4.7, Section 4.8, Section 4.9 and Section 4.10). For the analysis of VOCs emitted by shoots, an independent set of 20 plants per treatment was subjected to herbivory as described above, and VOCs were collected immediately after larval removal (Section 4.6.1).

The experiment consisted of four treatments: non-inoculated plants without herbivory (CONTROL:-Root Herbivory), non-inoculated plants exposed to larvae (CONTROL: +Root Herbivory), plants inoculated with *B. velezensis* GB03 without herbivory (PGPR: -Root Herbivory), and GB03-inoculated plants exposed to larvae (PGPR: +Root Herbivory). Seven days after transplanting, plants were inoculated by applying 1 mL of the bacterial suspension (10^8^ CFU mL^−1^) around the stem base, whereas control plants received 1 mL of sterile saline solution [51]. Each experiment included 60 plants per treatment (one plant per pot) arranged randomly in the growth chamber: 20 plants were used for biomass determination, 20 for chemical and molecular analyses and 20 for the collection of shoot VOCs. The experiment was conducted three times independently.

### 4.5. EO Extraction

EOs were extracted separately from leaves and roots. Previously weighed plant material from each organ was placed in a flask containing 50 mL of distilled water. EOs were extracted by hydrodistillation using a modified Clevenger apparatus for approximately 30 min. Following distillation, p-cymene (2 μL in 400 μL of methylene chloride) was added as an internal standard. p-cymene was selected because it is not a constituent of the EO of the plant under study and does not have a retention time similar to those of the major compounds analyzed. The distillate was then partitioned with 10 mL of methylene chloride, and the solvent was subsequently evaporated under reduced pressure at 40 °C. The EOs obtained separately from leaves and roots were analyzed by GC using a Trace 1300 gas chromatograph (Thermo Fisher Scientific, Waltham, MA, USA) equipped with a TG-5MS capillary column (30 m × 0.25 mm, 0.25 μm), following the method described by Banchio et al. [71].

This GC analysis enabled the quantitative determination of the EO constituents present in the samples obtained from the different treatments. The GC operating conditions were as follows: the oven temperature was initially maintained at 60 °C for 5 min and then increased to 240 °C at a rate of 20 °C min^−1^. The injector and detector temperatures were set at 250 °C. A flame ionization detector (FID) was used, with nitrogen as the carrier gas at a constant flow rate of 1.5 mL min^−1^. Compound identification was based on comparison of retention times with those of authentic standards (Sigma-Aldrich, St. Louis, MO, USA) analyzed under the same chromatographic conditions and was confirmed by GC-MS. GC-MS analyses were performed on a Clarus 580 gas chromatograph coupled to an SQ8 mass spectrometer (PerkinElmer, Waltham, MA, USA) equipped with an Elite-5MS (PerkinElmer) capillary column (30 m × 0.25 mm i.d., 0.25 μm film thickness). Helium was used as the carrier gas at 1 mL min^−1^, and the injector temperature was 250 °C. The oven temperature was held at 60 °C for 5 min, increased to 240 °C at 5 °C min^−1^ and held for 10 min. Samples (1 μL) were injected in split mode (1:50). Mass spectra were obtained by electron impact ionization at 70 eV in full-scan mode over *m*/*z* 50–350 (scan time 0.2 s, inter-scan delay 0.1 s) with a solvent delay of 5 min, using TurboMass 6.1.0 software. Compounds were identified by comparison of their mass spectra with those of the NIST library and of their retention indices with literature data.

### 4.6. Collection of Plant VOCs

VOCs emitted by shoots and roots of *O. basilicum* plants were collected separately using a dynamic headspace collection system with Super-Q adsorbent traps (30 mg, 80–100 mesh; Alltech Associates, Inc., Deerfield, IL, USA), following Cappellari et al. [54] with modifications. Prior to use, Super-Q traps were conditioned by washing with 5 mL of methylene chloride to remove potential impurities. p-Cymene was used as an internal standard because it was not emitted by the plants and did not have a retention time similar to those of the compounds analyzed. In both shoot and root VOC collections, a piece of filter paper impregnated with 10 μL of a 2% (*v*/*v*) p-cymene solution in methylene chloride was placed inside each collection chamber before sampling, so that the internal standard was trapped on the Super-Q adsorbent together with the plant volatiles. In both cases, VOCs were collected for 2 h under a continuous airflow of approximately 300 mL min^−1^. Immediately after collection, the Super-Q traps were removed and the retained compounds were eluted with 200 μL of methylene chloride. The resulting extracts were analyzed by GC using the same equipment, column and chromatographic conditions described for EO analysis, and compounds were identified as described in Section 4.5. Individual compounds were quantified by relating their peak areas to that of the internal standard, and total VOC emission was calculated as the sum of the peak areas of all detected plant-emitted compounds, excluding the internal standard and compounds detected in empty chambers. VOC emission was expressed as ng VOCs h^−1^ g^−1^ fresh weight.

#### 4.6.1. VOC Collection from Shoots

For the collection of VOCs emitted by shoots, individual plants from each treatment were placed in 1000-mL polyethylene terephthalate chambers. A rigid cover was placed over the pot surface to prevent the collection of volatile compounds potentially originating from the vermiculite substrate. After VOC collection, shoots were harvested and their fresh weight was determined. The major shoot VOCs quantified were 1,8-cineole, β-linalool, α-terpineol and eugenol.

#### 4.6.2. VOC Collection from Roots

Root VOCs were collected using a dynamic headspace approach adapted from Rasmann et al. [88]. Previously weighed roots were collected at harvest, immediately frozen in liquid nitrogen, and stored at −80 °C until analysis. Root samples were subsequently transferred to 100-mL Kitasato flasks, which served as VOC collection chambers and were sealed with perforated rubber stoppers to allow airflow through the system. The major root VOCs quantified were endo-borneol and eugenol.

### 4.7. Determination of Total Phenolic Content (TPC)

TPC was determined separately in leaves and roots using the Folin–Ciocalteu method [88]. Briefly, 150 mg of each tissue were homogenized with 5 mL of distilled water and allowed to stand for 24 h in the dark. An aliquot (0.5 mL) of each plant extract was then mixed with 8 mL of distilled water and 0.5 mL of Folin–Ciocalteu reagent. After 5 min, 1 mL of aqueous Na_2_CO_3_ (20%, *w*/*v*) was added. After 1 h, absorbance was measured at 760 nm using a Jenway 7305 spectrophotometer (Jenway, Stone, Staffordshire, UK). For quantification, a calibration curve was prepared using gallic acid as the standard phenolic compound. Total phenolic content was expressed as gallic acid equivalents per gram of fresh weight (FW) [68].

### 4.8. Total RNA Extraction and Quantitative Real-Time PCR

RNA extraction from leaf tissue of basil plants inoculated with rhizobacteria was performed according to the method described by Wang et al. [89]. Starting with 60 mg of freeze-dried plant material, the innuPREP Plant RNA Kit (Analytik Jena, Jena, Germany) was used for RNA extraction. A slight modification suggested by Kalinowska et al. [90] was incorporated: the samples were ground to a fine powder in a mortar using liquid nitrogen. The extracted RNA was verified and quantified using a NanoDrop 2000c spectrophotometer (Thermo Scientific, Wilmington, DE, USA). To degrade any remaining total DNA, an additional step involving DNAase incubation was included in the provided protocol. The resulting RNA was stored at −80 °C until further use. Reverse Transcription Polymerase Chain Reaction (RT-PCR) was used to synthesize complementary DNA (cDNA) from the obtained RNA samples. The Superscript III kit (Invitrogen, Carlsbad, CA, USA) was used to generate cDNA templates from 1 µg of total RNA. The experiment was conducted in a thermocycler, with a precise program. The RNA, oligonucleotides, and dNTPs were incubated together for five minutes at a temperature of 65 °C. The enzymes were added next, along with 5× First-Strand Buffer and 0.1 M DTT. The mixture was then incubated for one hour at 55 °C. Finally, the reaction was inactivated by heating it to 70 °C for 15 min. The resulting cDNA was then diluted in 80 μL of PCR-grade water and stored at −20 °C for later use.

Quantitative real-time PCR (qPCR) was performed using EvaGreen^®^ No Rox qPCR Mix (Bio&Sell, Feucht, Germany) to assess the expression of genes related to phenylpropanoid and eugenol biosynthesis, *EGS* (eugenol synthase) and *C4H* (cinnamate 4-hydroxylase). Actin was selected as the reference gene because its stable expression in *O. basilicum* under stress conditions has been reported previously [91] and are listed in Table 5. All samples were analyzed in triplicate on 96-well plates. The reaction mixture contained 2 μL of cDNA, 0.5 μL of each primer, and 5 μL of PCR-grade water, in a final volume of 10 μL. Reactions were performed on a CFX Connect™ Real-Time PCR Detection System (Bio-Rad, Munich, Germany) under the following conditions: 95 °C for 3 min, followed by 50 cycles of 95 °C for 10 s and 60 °C for 20 s. Melt curve analysis was performed at the end of each run, and no-template controls were included. To ensure analytical reliability, primer amplification efficiencies were determined from a five-fold serial dilution of pooled cDNA. All primer pairs (actin, EGS, C4H) showed efficiencies between 90–110% with correlation coefficients (R2) > 0.99. Actin was selected as the reference gene because its stable expression in *O. basilicum* under comparable experimental conditions has been reported previously [91,92]. In addition, actin stability across treatments was confirmed by evaluating Ct variation (<0.5 Ct), ensuring reliable normalization.

Relative gene expression was calculated using the 2−ΔΔCt method, following Livak & Schmittgen et al. [93], based on three biological replicates, each analyzed with three technical replicates.

### 4.9. Hormone Extraction

Phytohormones were extracted from 50 mg of freeze-dried, homogenized leaf material using three rounds of extraction in 80% methanol, which was acidified to pH 2.4 with hydrochloric acid. For round 1, 400 µL, for round 2, 200 µL, and, for round 3, 100 µL solvent was used. Cell extraction was performed in 1.5 mL cryo-tubes with reinforced walls (Biozyme, Hessisch Oldendorf, Germany). In order to enhance cell rupture and extraction, one steel bead of 3 mm, three steel beads of 1 mm diameter, and glass beads of 0.75 to 1 mm diameter (Carl Roth, Karlsruhe, Germany) were added to each tube, and bead milling was performed for 3 × 1 min in a homogenizer (FastPrep24, MP Biomedicals, Santa Ana, CA, USA). The extraction solvent contained known amounts of deuterated internal standards ([2H6]-ABA, [2H6]-JA, [2H2]-JA-Ile, [2H5]-OPDA and [2H4]-SA) [94].

### 4.10. SPE-UPLC-MS/MS Phytohormone Analysis

Phytohormones were separated using a Nucleoshell RP Biphenyl column (100 mm × 2 mm, 2.1 µm, Macherey und Nagel, Düren, Germany) with the following gradient: 0–2 min: 5% B; 13 min: 95% B; 13–15 min: 95% B; 15–18 min: 5% B. The column temperature was set to 40 °C. Solvent A consisted of 0.3 mM ammonium formate, acidified with formic acid to pH 3.0, while solvent B was acetonitrile. The autosampler temperature was maintained at 4 °C. For sample preparation, a prototype solid-phase extraction (SPE) system was used in conjunction with UPLC. Per sample, 600 µL of plant extract was injected onto a divinylbenzene micro-SPE stationary phase (30 mg, SparkHolland B.V., Emmen, The Netherlands) at a flow rate of 200 µL min^−1^. Phytohormones were retained by the simultaneous addition of excess water (3800 µL min^−1^). Transfer from the SPE cartridge to the UPLC column was performed using 120 µL of a solution containing 20% acetonitrile and 80% solvent A. The entire procedure was carried out on a prototype system composed of a CTC Combi-PAL autosampler (1 mL injection loop), an ACE 96-well plate SPE unit, a high-pressure dispenser, an SPH1299 UPLC gradient pump, an EPH30 UPLC dilution pump, and a Mitral column oven (all from Axel-Sembrau GmbH, Sprockhövel, Germany). Phytohormones were detected by mass spectrometry using a QTrap 6500 (Sciex, Framingham, MA, USA) with electrospray ionization in positive mode. Detection was performed by multiple reaction monitoring (MRM) with a 5 ms dwell time per transition. The ion source was heated to 450 °C. The curtain gas was set to 35 psi, while ion source gases GS1 and GS2 were set to 60 psi and 70 psi, respectively. The electrospray ion spray voltage was 5500 V.

### 4.11. Statistical Analysis

Normality and homoscedasticity of the data were assessed using the Shapiro–Wilk and Levene tests, respectively. Data were analyzed by two-way analysis of variance (ANOVA), with GB03 inoculation and root herbivory as the two factors. When significant differences were detected, treatment means were compared using Fisher’s least significant difference (LSD) post hoc test. Differences were considered statistically significant at *p* < 0.05. Principal component analysis (PCA) was performed as an exploratory analysis on treatment means of the measured variables, standardized to unit variance, to visualize the overall response patterns among treatments. All statistical analyses were performed using InfoStat software, version 2020 (InfoStat Group, Universidad Nacional de Córdoba, Córdoba, Argentina).

## 5. Conclusions

*B. velezensis* GB03 and root herbivory by *D. speciosa* induced distinct but interconnected responses in *O. basilicum*. GB03 promoted plant growth and modified basal specialized metabolism, whereas root herbivory reduced biomass, activated jasmonate-related signaling, and induced metabolic changes in both roots and shoots. When both factors occurred together, several components of specialized metabolism reached their highest levels, particularly those related to essential oils, phenolic compounds, VOCs, and eugenol metabolism. Importantly, these responses did not represent a generalized enhancement of plant defense. The effects of herbivory on shoot phenolic accumulation, root VOC emission, and JA depended on the inoculation status of the plants, demonstrating that GB03 selectively modulates specific local and systemic responses to belowground herbivory. Considered together with our previous studies using the same *O. basilicum*–GB03 system with foliar-chewing and phloem-feeding herbivores, the present findings further indicate that GB03-mediated modulation of plant responses is strongly influenced by herbivore identity, feeding strategy, and site of attack. Thus, GB03 appears to establish a distinct physiological and metabolic context from which basil responds differently to subsequent herbivore challenges. These findings highlight the plasticity of plant–PGPR–herbivore interactions and the importance of considering the specific biological context when evaluating PGPR-mediated effects on plant defense. The capacity of GB03 to promote plant growth while reshaping specialized metabolism under herbivore pressure supports further investigation of beneficial rhizobacteria as tools for sustainable management of aromatic crops. Future studies evaluating insect performance and preference under more complex and field-relevant conditions will be important to determine whether these metabolic and signaling changes translate into increased resistance to *D. speciosa*.

## Figures and Tables

**Figure 1:**
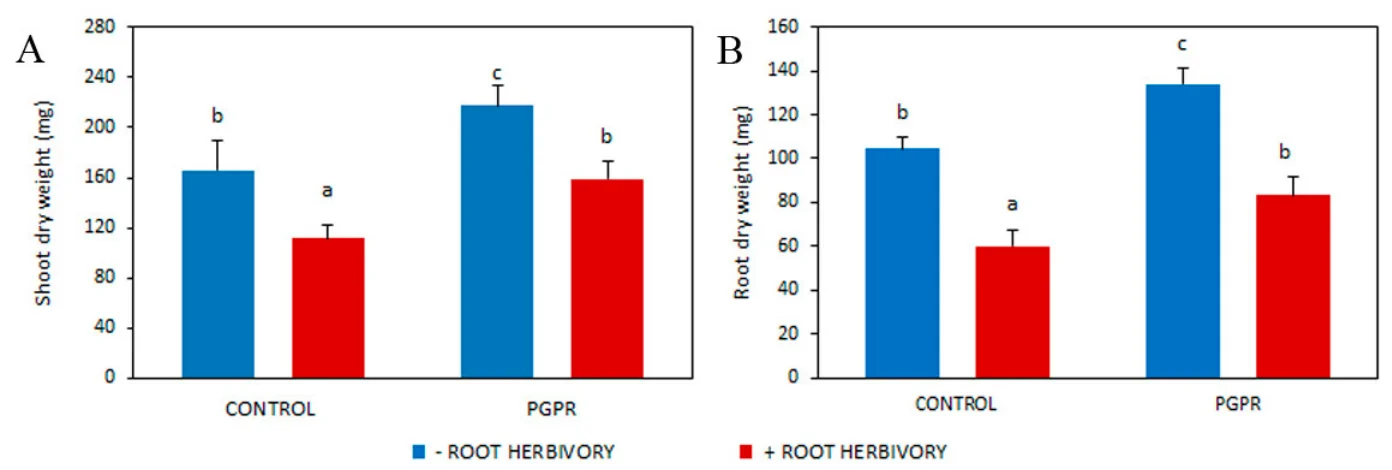
Shoot dry weight (**A**) and root dry weight (**B**) in *O. basilicum* plants inoculated with the PGPR strain *B. velezensis* GB03 and/or subjected to root herbivory. Values are means ± SE (n = 15). Different letters above bars indicate significant differences according to Fisher’s LSD test (*p* < 0.05).

**Figure 2:**
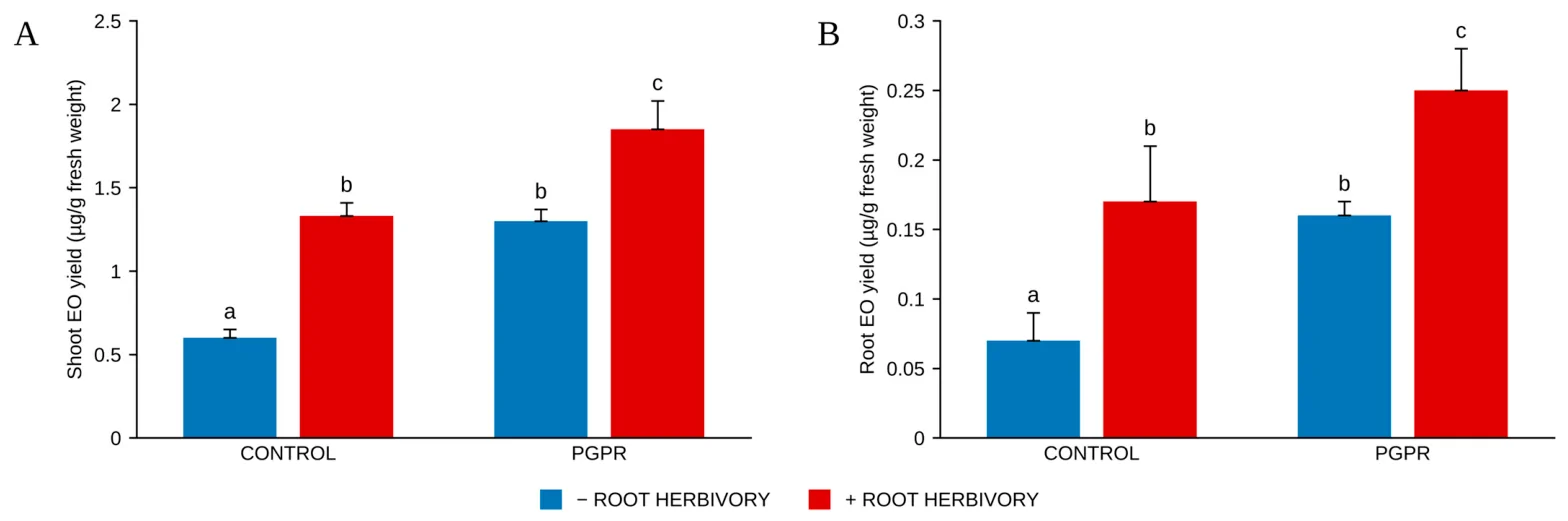
EO yield in shoot (**A**) and root (**B**) organs of *O. basilicum* plants inoculated with the PGPR strain *B. velezensis* GB03 and/or subjected to root herbivory. Values are means ± standard error (SE) (n = 20). Different letters above bars indicate significant differences according to Fisher’s LSD test (*p* < 0.05).

**Figure 3:**
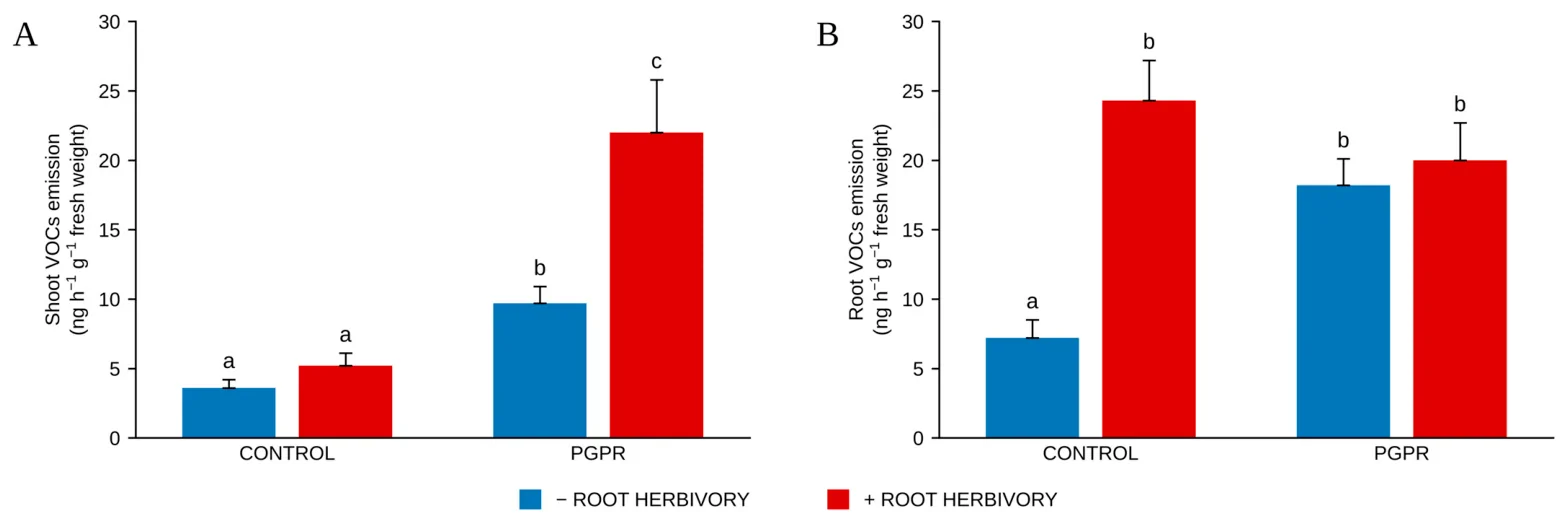
Volatile organic compound (VOC) emission in shoots (**A**) and roots (**B**) of *Ocimum basilicum* plants inoculated with the PGPR strain *Bacillus velezensis* GB03 and/or subjected to root herbivory by *Diabrotica speciosa*. Values are means ± standard error (SE) (n = 15). Different letters above bars indicate significant differences among treatments according to Fisher’s LSD test (*p* < 0.05).

**Figure 4:**
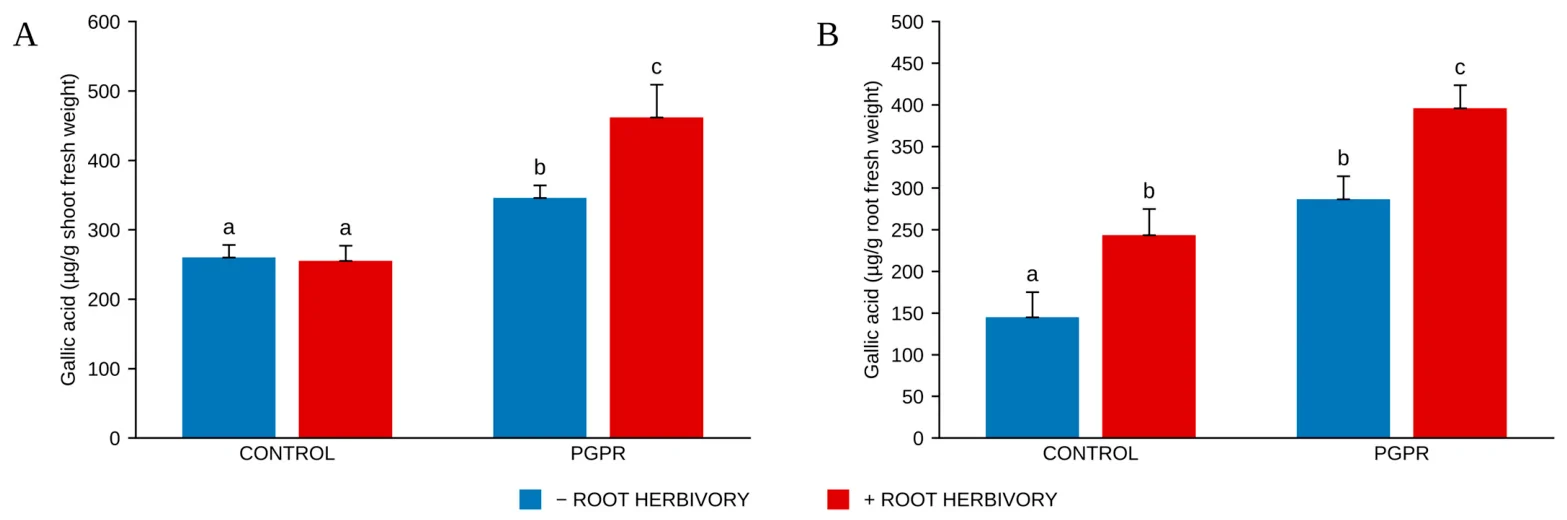
Total phenolic content (TPC) in shoots (**A**) and roots (**B**) of *Ocimum basilicum* plants inoculated with the PGPR strain *Bacillus velezensis* GB03 and/or subjected to root herbivory by *Diabrotica speciosa*. Values are means ± standard error (SE) (n = 20). Different letters above bars indicate significant differences among treatments according to Fisher’s LSD test (*p* < 0.05).

**Figure 5:**
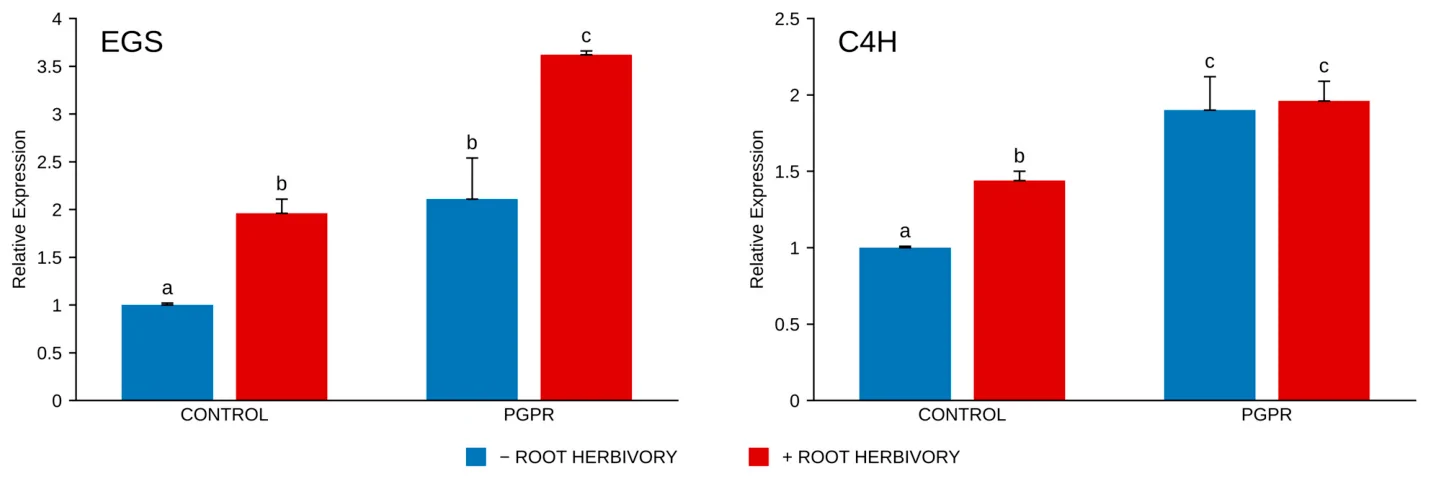
Relative expression of *EGS* and *C4H* genes in *Ocimum basilicum* plants inoculated with the PGPR strain *Bacillus velezensis* GB03 and/or subjected to root herbivory by *Diabrotica speciosa*. Values are means ± standard error (SE) (n = 3). Different letters indicate significant differences among treatments according to Fisher’s LSD test (*p* < 0.05).

**Figure 6:**
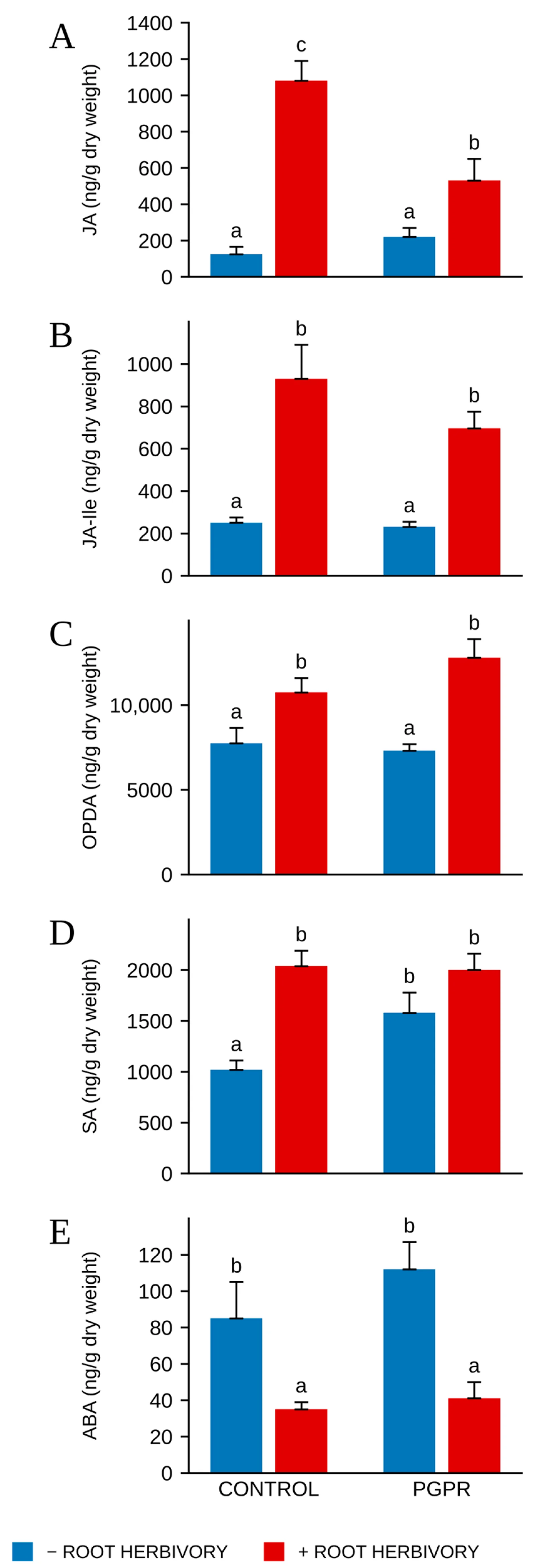
Endogenous levels of (**A**) jasmonic acid (JA), (**B**) jasmonoyl-L-isoleucine (JA-Ile), (**C**) 12-oxo-phytodienoic acid (OPDA), (**D**) salicylic acid (SA), and (**E**) abscisic acid (ABA) in *O. basilicum* plants inoculated with *B. velezensis* GB03 and/or subjected to root herbivory by *D. speciosa*. Values are means ± SE. Different letters indicate significant differences among treatments according to Fisher’s LSD test (*p* < 0.05).

**Figure 7:**
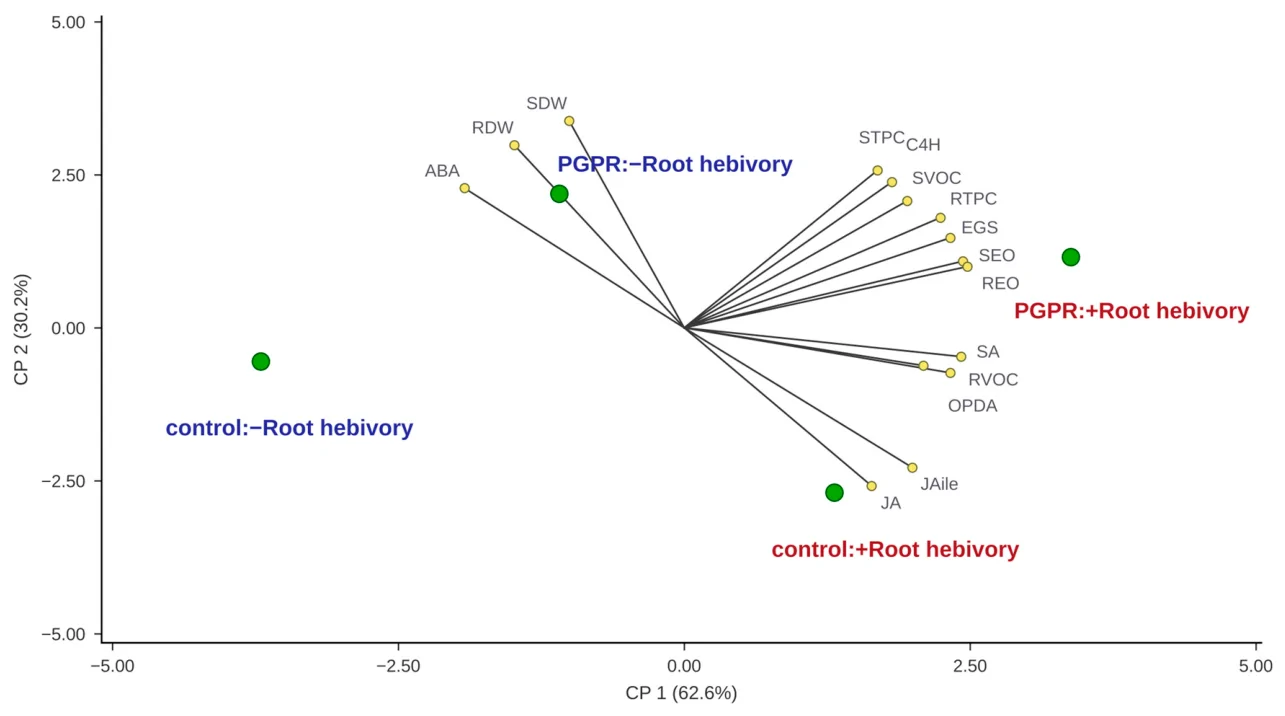
Principal component analysis (PCA) based on treatment means of growth, hormonal, molecular, and specialized-metabolism responses of *O. basilicum* plants inoculated with *B. velezensis* GB03 and/or subjected to root herbivory by *D. speciosa*. The first two principal components (PC1 and PC2) explained 62.6% and 30.2% of the total variance, respectively. Treatments are represented by green circles and variables by vectors. SDW, shoot dry weight; RDW, root dry weight; ABA, abscisic acid; SA, salicylic acid; JA, jasmonic acid; JA-Ile, jasmonoyl-L-isoleucine; OPDA, 12-oxo-phytodienoic acid; STPC, shoot total phenolic content; RTPC, root total phenolic content; SVOCs, shoot volatile organic compounds; RVOCs, root volatile organic compounds; SEO, shoot essential oil; REO, root essential oil; EGS, eugenol synthase; C4H, cinnamate 4-hydroxylase.

**Table 1:** Concentrations (µg/g fresh weight) of major essential oil (EO) components in shoots of *O. basilicum* plants inoculated with the PGPR strain *B. velezensis* GB03 and/or subjected to root herbivory by *D. speciosa*. Values are means ± standard error (SE). Different letters indicate significant differences according to Fisher’s LSD test (*p* < 0.05).

Treatment	1,8-Cineole	Linalool	Terpineol	Eugenol
Control				
−Root herbivory	0.08 ± 0.01 a	0.08 ± 0.01 a	0.01 ± 0.001 a	0.35 ± 0.02 a
+Root herbivory	0.16 ± 0.02 bc	0.24 ± 0.04 b	0.03 ± 0.003 bc	1.06 ± 0.11 c
*PGPR*				
−Root herbivory	0.12 ± 0.01 b	0.24 ± 0.03 b	0.02 ± 0.001 b	0.78 ± 0.04 b
+Root herbivory	0.18 ± 0.01 c	0.28 ± 0.03 b	0.03 ± 0.001 c	1.33 ± 0.12 d

**Table 2:** Concentrations (µg/g fresh weight) of major essential oil (EO) components in roots of *O. basilicum* plants inoculated with the PGPR strain *B. velezensis* GB03 and/or subjected to root herbivory by D. speciosa. Values are means ± standard error (SE). Different letters indicate significant differences according to Fisher’s LSD test (*p* < 0.05).

Treatment	Endo-Borneol	Eugenol
Control		
−Root herbivory	0.01 ± 0.002 ab	0.01 ± 0.001 a
+Root herbivory	0.01 ± 0.001 a	0.05 ± 0.01 b
*PGPR*		
−Root herbivory	0.01 ± 0.001 ab	0.07 ± 0.01 b
+Root herbivory	0.03 ± 0.01 b	0.12 ± 0.02 c

**Table 3:** Emission rates (ng h^−1^ g^−1^ FW) of major VOCs from shoots of *O. basilicum* plants inoculated with the PGPR strain *B. velezensis* GB03 and/or subjected to root herbivory by *D. speciosa*. Values are means ± standard error (SE). Different letters indicate significant differences according to Fisher’s LSD test (*p* < 0.05).

Treatment	Cineole	Linalool	Terpineol	Eugenol
Control				
−Root herbivory	0.19 ± 0.04 a	0.41 ± 0.09 a	0.12 ± 0.03 a	0.14 ± 0.04 a
+Root herbivory	0.30 ± 0.06 ab	0.65 ± 0.13 ab	0.42 ± 0.09 b	0.40 ± 0.06 a
*Rhizobacteria*				
−Root herbivory	0.33 ± 0.06 ab	1.02 ± 0.15 b	0.36 ± 0.09 ab	0.68 ± 0.12 b
+Root herbivory	0.40 ± 0.06 b	1.78 ± 0.26 c	0.97 ± 0.13 c	0.72 ± 0.14 b

**Table 4:** Emission rates (ng h^−1^ g^−1^ FW) of major VOCs from roots of *O. basilicum* plants inoculated with the PGPR strain *B. velezensis* GB03 and/or subjected to root herbivory by *D. speciosa*. Values are means ± standard error (SE). Different letters indicate significant differences according to Fisher’s LSD test (*p* < 0.05).

Treatment	Endo-Borneol	Eugenol
Control		
−Root herbivory	1.28 ± 0.16 a	2.08 ± 0.27 a
+Root herbivory	3.98 ± 0.58 b	2.97 ± 0.39 ab
PGPR		
−Root herbivory	3.20 ± 0.44 b	3.65 ± 0.77 b
+Root herbivory	2.81 ± 0.37 b	3.66 ± 0.49 b

**Table 5:** Primer sequences for RT-PCR.

Gene	Forward Primer Sequence (5′-3′)	Reverse Primer Sequence (5′-3′)
Actin	AGATTCCTCCAGCAAATCTTTCTC	CTTTCTGGTGGAACAGCATCAA
EGS	ACCCATAGCAATCCTTCACTG	AGTTGAAGCCTCCACATCGT
C4H	GCCAACAACCCCGCTCAATG	CCAACGCCGAAGGGGAGGTATC

## Data Availability

The data presented in this study are available on request from the corresponding author.
